# Microgravity × Radiation: A Space Mechanobiology Approach Toward Cardiovascular Function and Disease

**DOI:** 10.3389/fcell.2021.750775

**Published:** 2021-10-29

**Authors:** Carin Basirun, Melanie L. Ferlazzo, Nicholas R. Howell, Guo-Jun Liu, Ryan J. Middleton, Boris Martinac, S. Anand Narayanan, Kate Poole, Carmine Gentile, Joshua Chou

**Affiliations:** ^1^School of Biomedical Engineering, Faculty of Engineering and Information Technology, University of Technology Sydney, Sydney, NSW, Australia; ^2^Australian Nuclear Science and Technology Organisation, Lucas Heights, NSW, Australia; ^3^Inserm, U1296 Unit, Radiation: Defense, Health and Environment, Centre Léon Bérard, Lyon, France; ^4^Discipline of Medical Imaging and Radiation Sciences, Faculty of Medicine and Health, Brain and Mind Centre, The University of Sydney, Camperdown, NSW, Australia; ^5^Molecular Cardiology and Biophysics Division, Victor Chang Cardiac Research Institute, Sydney, NSW, Australia; ^6^Department of Nutrition and Integrative Physiology, Florida State University, Tallahassee, FL, United States; ^7^EMBL Australia Node in Single Molecule Science, Faculty of Medicine, School of Medical Sciences, University of New South Wales, Sydney, NSW, Australia; ^8^Faculty of Medicine and Health, Sydney Medical School, The University of Sydney, Sydney, NSW, Australia

**Keywords:** mechanobiology, microgravity, cardiovascular, mechanotransduction, cardiac disease, radiation

## Abstract

In recent years, there has been an increasing interest in space exploration, supported by the accelerated technological advancements in the field. This has led to a new potential environment that humans could be exposed to in the very near future, and therefore an increasing request to evaluate the impact this may have on our body, including health risks associated with this endeavor. A critical component in regulating the human pathophysiology is represented by the cardiovascular system, which may be heavily affected in these extreme environments of microgravity and radiation. This mini review aims to identify the impact of microgravity and radiation on the cardiovascular system. Being able to understand the effect that comes with deep space explorations, including that of microgravity and space radiation, may also allow us to get a deeper understanding of the heart and ultimately our own basic physiological processes. This information may unlock new factors to consider with space exploration whilst simultaneously increasing our knowledge of the cardiovascular system and potentially associated diseases.

## Introduction

Cardiovascular disease (CVD) is one of the leading causes of death in adults in many countries (Lozano et al., 2012). Due to the devastating rate of fatality, many researchers have been motivated to develop clinical models to simulate responses to treatment and drugs to CVD (Gentile, 2016; Polonchuk et al., 2017, 2021; Roche et al., 2021; Sharma and Gentile, 2021; Sharma et al., 2021). New approach and concept are needed to understand the complexity of cardiac function and disease onset. Interestingly, the effects of spaceflight on the human body appear to mimic an accelerated aging process that negatively impacts the heart and the cardiovascular system and closely resembles CVD (Hughson et al., 2016, 2018). Furthermore, the heart is also known to be sensitive to radiation; therefore, the response and any associated risk and diseases need to be considered when examining the impact of space exploration. As such, a deeper understanding of the cardiogenic effects of microgravity and radiation may have implications for human spaceflight as well as treat millions of heart disease patients on Earth. As microgravity and radiation happen simultaneously during space travel, it is of great interest to study the compounding impact on living matter exposed to microgravity and radiation environment at the same time. Due to the complexity of such research, there is still much unknown regarding the effect of spaceflight on the cardiovascular system. In this mini review, a new approach toward the understanding of cardiac function and disease can be found in space mechanobiology. Space mechanobiology can be defined as the study of how cells are influenced by their physical environment and in this case by the microgravity environment. To fully understand the function and underlying mechanisms of cardiac function and the mechanism and markers for CVD, it is important to first understand how cardiac cells transduce these mechanical stimuli. The extracellular matrix (ECM) plays a vital part in carrying and providing mechanical cues to these cardiac cells. These mechanical cues are then transformed into biological responses (Martino et al., 2018). As the heart is a mechanical organ that is highly responsive to different types of mechanical stimuli (Takahashi et al., 2013; Guo and Huebsch, 2020), this mini review will demonstrate the importance of mechanotransduction and how space microgravity and radiation can be leveraged to advance our understanding of cardiac function and disease.

## The Effect of Microgravity on the Cardiovascular System

Exposure to microgravity which differs from the normal state of gravity, puts the human body under critical physiological changes, some of the considerable importance to survival. Gravity is an important factor of fluid distribution and therefore plays an important factor in the cardiovascular system. Under normal gravity where the human body is in an upright position, there is higher arterial pressure (200 mmHg) in the feet and lower pressure (70 mmHg) in the head. Whereas the heart relative to arterial pressure is 100 mmHg. In a microgravity environment this fluid distribution is altered, losing the gradient and the fluid distribution becomes more uniform throughout the body (Hargens and Richardson, 2009; Demontis et al., 2017). This, in turn, reduces the demand for arterial blood pressure and decreases the amount of work the heart has to do leading to cardiovascular deconditioning and increased risk of CVD.

Head-down tilt is one ground-based analog model for studying simulated microgravity and therefore cardiovascular deconditioning effects, where previous reports utilizing this model have shown a significant elevation of microvascular pressures in the head, and increased capillary fluid filtration (Parazynski et al., 1991; Christ et al., 2001). Short-duration spaceflight has shown increased capillary fluid filtration, as well; however, this phenomenon is unknown in the context of long-duration spaceflight (Leach et al., 1996). These microvascular adaptations are related with the fluid redistribution phenomenon highlighted previously, and in turn, suggest vascular adaptations. Several *ex vivo* studies have shown that the structure and function of blood vessels certainly adapts to spaceflight, real and simulated, however, there currently exist no *in vivo* rodent equivalent studies (Delp, 1999, 2007). There exists a much more thorough literature of *in vivo* studies, both of crew and rodent analog models, spaceflight and ground based. Observations from astronauts that have returned from a 6-month spaceflight have shown an increase in stiffness in the carotid artery, which is comparable to 20 years of aging (Kawasaki et al., 1987; Gepner et al., 2014; Hughson et al., 2016). This reduced cardiac function leads to a reduction in tissue mass of the heart, also known as cardiac atrophy, which ultimately causes debilitating changes in heart function. More recent *in vivo* vascular studies of crew during long-duration International Space Station (ISS) missions have shown spaceflight-induced structural and functional changes of the carotid and brachial arteries. From this study, no changes in carotid or brachial arteries were generally seen; however, it is important to note that crew flight studies have a small sample size and variability between crew responses that may influence certain measures. For indeed in this study, cardiovascular biomarkers of oxidative stress and inflammation (e.g., increased PGF2a, oxidized LDL, TNF-a, myeloperoxidase, etc.) were elevated during spaceflight (Lee et al., 2020). Indeed, there are suggestions that all astronauts more notably the Apollo astronauts that traveled to the Moon and back had comparatively greater CVD risks (Delp et al., 2016).

Although it remains unclear how future exploration missions, and as a result, extended altered gravity field durations and higher radiation exposures may exacerbate these effects. On-going and future studies and further *in vivo* rodent studies complemented with *ex vivo* and *in vitro* analyses to assess the functional, structural, and biochemical adaptations of cardiovascular adaptations to spaceflight can be utilized in the field of space mechanobiology for more in-depth study for the cardiovascular system.

## The Role(s) Played by Microgravity on Cardiomyocyte Function

Spaceflight exposure also leads to various cellular and organ adaptations, though the *in vivo* study of cardiovascular adaptations, whether through actual and/or ground simulated studies, is limited. Several studies have looked at the impact of microgravity on cell proliferation and differentiation. These studies have demonstrated that stem cells that were grown in a microgravity environment grow differently from those grown under normal gravity, showing changes in division, contraction, and migration (Zhang et al., 2015; Shinde et al., 2016).

Recent studies that involved the cells of the cardiovascular system looked at cardiac progenitor cells (CPCs) cultured on the ISS. Alterations to transcriptional control were measured and it was found that among both neonatal and adult CPCs, the significantly dysregulated microRNAs affect cytoskeletal remodeling and mechanotransduction pathways. Genes related to mechanotransduction were downregulated, while the expression of cytoskeletal genes and calcium signaling molecules was significantly elevated only in neonatal CPCs. Cytoskeletal organization and migration were both affected by spaceflight in neonatal and adult CPCs, however, only neonatal CPCs experienced increased expression of early developmental markers and a higher potential to proliferate (Baio et al., 2018; Camberos et al., 2019, 2021).

Wnorowski et al. (2019) have shown that monolayers of beating cardiomyocytes derived from human induced pluripotent stem cells (hiPSC-CMs) that were sent to the ISS for a period of 5.5 weeks, had no significant effect on the morphology. Looking at functional differences between the two samples, there were no significant difference in velocity during contraction and relaxation. However, some functional differences were observed, where the space-flown hiPSC-CMs had a decreased calcium recycling rate as well as an observed beating irregularity when the samples were assessed following their return to Earth. These results suggested that following the samples return to normal gravity, their calcium-handling-related parameters remained altered (Wnorowski et al., 2019).

Although Baio et al. (2018) and Wnorowski et al. (2019) had similar microgravity conditions with the samples flown to the ISS, there were discrepancies in results including the contradiction in the expression of DNA repair genes and this may be attributed by several experimental conditions, one of them being that the type of cells used by Baio et al. (2018) being progenitor cells and Wnorowski et al. (2019) used differentiated cardiomyocytes. There are other experimental conditions that can be considered between these and other experiments in the field, including the duration of exposure to microgravity and the way the cells were processed for the assays. Other studies including different cell types in the cardiovascular response to simulated microgravity is summarized in Table 1A and the response to spaceflight microgravity is summarized in Table 1B.

**TABLE 1:** Summary of cardiovascular responses to microgravity and radiation.

**(A) Microgravity-induced cardiovascular response**				
**Cell origin**	**μG simulation method**	**Findings**		**References**
Human pluripotent stem cells (hPSCs)	Random positioning machine (RPM)	- Production of highly viable and enriched cardiomyocytes		
		- Promote the induction of cardiac progenitors, CM differentiation, proliferation and		Jha et al., 2016
		- increase cell survival of cardiac progenitors		
Endothelial cells (EA.hy926)	Random positioning machine (RPM)	- Increase in ECM proteins and altered cytoskeletal components and intermediate filaments		Infanger et al., 2006
		- Induced apoptosis in endothelial cells		
		- Formation of 3D cell aggregates; assembly to tube like structures		
Human pulmonary microvascular endothelial cells (HPMECs)	3D clinostat	- Tight junctions between cells were absent		Kang et al., 2011
		- Apoptosis positive cells are extensively shown		
		- Disorganized and depolymerized, extenuated actin filaments		
Heart cells from 2–4-day old rats and 15-day old chick embryos	Rotating bioreactor	- Rat heart cells-based construct observed spontaneous and synchronous contraction		Freed and Vunjak-Novakovic, 1997
		- Highest fraction of total regenerated tissue mass		
HL-1 cardiomyocytes	2D clinostat	- Increased concentrations of cytosolic calcium and spontaneous calcium oscillations		
		- Induced cardiomyocyte atrophy		Liu et al., 2020
		- Decreased cell size		
Primary rat neonatal cardiomyocytes (NRCM)	Rotating wall vessel (RWV)	- Decreased protein turnover		
		- Unaffected apoptosis, cell viability and protein degradation		
		- Upregulation of protein content and processes in mitochondrial protein translation		Feger et al., 2016
		- Downregulation in proteins and protein translation in the rough endoplasmic reticulum and ribosomes		
Adult and neonatal cardiac progenitor cells (Isl-1 + CPCs)	2D clinostat	- Age dependent responses tube formation		Fuentes et al., 2015
		- Increase in growth factor expression		
		- Elevated expression of stemness associated genes in neonatal CPCs		
		- Elevated transcription of DNA repair genes in neonatal CPCs		
Endothelial cells (EA.hy926)	Random positioning machine (RPM)	- Formation of tube-like structure with walls of single-layered ECs		Grimm et al., 2009

**(B) Effect of microgravity and radiation on cardiovascular system cells**				
***in vitro***				
**Cell origin**	**Space μG/μG simulation method**	**Space radiation/radiation source**	**Findings**	**References**

Human induced pluripotent stem cells – derived cardiomyocytes (hiPSC-CMs)	Space μG (ISS)	Space radiation (ISS)	- Decreased calcium recycling rate as well as an observed beating irregularity - Significant upregulation of sarcomere genes - Significant decrease in DNA damage and repair genes - 2,635 genes were differentially expressed among flight, post-flight, and ground control samples	Wnorowski et al., 2019
Adult cardiac progenitor cells (CPCs)	Space μG (ISS)/Simulated μG (2D clinostat)	Space radiation (ISS)	- Upregulation of YAP1 expression - Short term YAP1 activation	Camberos et al., 2019
Neonatal and adult human cardiac progenitor cells (CPCs)	Space μG (ISS)	Space radiation (ISS)	- Mechanotransduction genes are downregulated in neonatal CPCs and upregulated in adult CPCs - Increased expression of early developmental markers and enhanced proliferative potential in neonatal CPCs - Increased expression of DNA repair genes in both adult and neonatal CPCs	Baio et al., 2018
Adult and neonatal cardiac progenitor cells (CPCs)	Space μG (ISS)	Space radiation (ISS)	- Transcript associated with stemness is significantly elevated - Key transcripts involved in cell cycle progression, cell differentiation, heart development, oxidative stress and focal adhesion were induced	Camberos et al., 2021

**(C) Radiation-induced cardiovascular response**				
**Cell origin**	**Radiation source**	**Findings**		**References**

Wistar (Harlan) male rats	HZE 56Fe-ion (0, 0.5, or 1 Gy)	- *In vivo* aortic stiffness		
		- Significantly higher ROS production		Soucy et al., 2011
Human umbilical vein cells (HUVEC)	Iron-ion or proton (1 Gy)	- Inhibition of vessel formation		Grabham et al., 2013
C57BL/6J mice	Proton (0.1 Gy) or heavy iron ions (0.5 Gy)	- Alternations in the methylation and expression of DNA repetitive elements in cardiac tissues		Koturbash et al., 2016

## The Effect of Radiation on the Cardiovascular System

Space radiation consists of solar and cosmic galactic rays (CGR) mainly composed of respectively, low and high energy protons, alpha particles and a minority of heavy charged particles and nuclei. Solar Particle Events (SPE) are temporary phenomena that increase the flux of protons. Astronauts are exposed to these solar and cosmic components only during Extra Vehicular Activities (EVA) and most of the times they remain inside their spacecraft or the ISS, where SPE could still be considered. Another source of radiation is the interaction of solar and CGR with shielding, creating high Linear Energy Transfer (LET) but low energy metallic particles and protons. The inside of the vessel is also subjected to a higher constant low dose of gamma radiation, about twice the highest natural radiation background found on Earth (Restier-Verlet et al., 2021).

When discussing radiation, we measure the “activity” of radiation where we measure how much radiation is coming out of something and “exposure” to radiation that measure the effect of radiation that has been absorbed by the substance. Radiation exposure is measured in gray (Gy), and this allows for a common unit of measurement of different types of radiation by measuring their effect on materials. Since not all radiation has the same effect biologically, we use sieverts (Sv) to measure the absorbed dose in human tissue to the biological damage to radiation (Australian Radiation Protection and Nuclear Safety Agency [ARPANSA], n.d.). The radiation doses received for space explorers depend on the time and distance from earth. Estimated doses are 150 mSv/year for space flight in the ISS, 300–400 mSv for traveling to the Moon, and 300–400 mSv/year and 1600–2300 mSv for 2 years traveling to Mars (Shavers et al., 2004; Chancellor et al., 2018; Restier-Verlet et al., 2021) in higher doses of radiation (>100 mGy) it has been clinically shown to lead to cardiac dysfunction over time, where cases have been reported following radiotherapy. Short and long-term effects of radiation induced cardiovascular diseases were observed following radiotherapy treatment of cancers adjacent to heart including lungs, breasts, and esophagus. The hearts of patients received 1.6–3.9 Gy radiation during radiotherapy treatment of peptic ulcer disease increased risk of coronary heart disease (Carr et al., 2005). In breast cancer radiotherapy, women’s hearts received doses 2.7–6.3 Gy which significantly increased risk of developing ischemic heart disease (McGale et al., 2011). Hughson et al. (2016) studies have suggested that astronauts spending 6 months in spaceflight increased stiffness of carotid artery and insulin resistance. The increase stiffness of carotid artery and insulin resistance may cause by multiple and compounding factors including radiation and microgravity. In a rat model Soucy et al. (2011) have demonstrated the detrimental effects of ionizing radiation. The rats exposed 1 Gy high-energy iron-ion radiation at approximately 0.5 Gy/min exhibit higher aortic stiffness and a greater level of endothelial dysfunction 4 months post radiation. This mainly involves increased oxidative stress as well as inflammation, playing a role in radiation-induced cardiovascular damage, which has been highlighted by multiple studies (Soucy et al., 2011; Coleman et al., 2015; Seawright et al., 2017; Table 1C). The relationship between dosage and response for some biological effects are therefore not linear and not proportional to dose (Betlazar et al., 2016, 2020, 2021; Puukila et al., 2017; Tang and Loganovsky, 2018). Chronic low doses of radiation (<100 mGy) may not be detrimental and could even induce benefits to the central nervous system and possibly to the cardiovascular system. However, it is unknown what impact of primarily low-dose rate radiation will have on the cardiovascular system during space exploration (Boerma et al., 2016), particularly in the presence of compounding factors including microgravity. Therefore, the study by combing compounding factors radiation and microgravity is crucial to examine the impact of space exploration on the cardiovascular system.

Microgravity and space radiation are unique and extreme environments which have been demonstrated to induce or model onsets of cardiovascular diseases and therefore has the potential to elucidate the underlying mechanisms of cardiovascular biology. By using microgravity and radiation as “tools” we could increase our understanding in the mechanotransduction, function and mechanisms of the cardiovascular system and subsequently the on-set of diseases.

## Is it Microgravity or Radiation, or Both?

The majority of studies have focused on utilizing stem cells and looking into their development (Table 1B). There are currently limited studies that have explored the effects of both microgravity and space radiation as a mechanism for CVD-causing processes. Both microgravity and space radiation, separately, have been shown to exhibit significant changes at both subcellular and tissue levels; however, exploring the effects of the two stressors in combination would be beneficial to our understanding of the space environment.

Endothelial cells (ECs) have been observed to form tube-like aggregates during both spaceflight and simulated gravity, confirming the influence of microgravity for 3D aggregation of ECs with and without the influence of radiation (Krüger et al., 2019). Tan et al. (2020) looked at human bronchial epithelial Beas-2B cells under simulated microgravity for 48 h followed by X-ray radiation. Although this is not specific to the cardiovascular system, the results were interesting in that the effects were shown to be additive. This study showed that the simulated microgravity on its own significantly reduced the survival of the cells and with the addition of radiation (1–6 Gy) it further inhibited cell survival -and cell proliferation (Tan et al., 2020). It is also well known that DNA double-strand breaks (DSBs) are a prominent consequence of radiation exposure, forming γH2AX foci as an early cellular response to DSBs (Sedelnikova et al., 2007; Mah et al., 2010). In this study, it was observed that the compound effect of simulated microgravity and radiation significantly promoted the formation of γH2AX foci compared to independent exposure of the two factors (Tan et al., 2020). Looking into studies done on the ISS, Wnorowski et al. (2019) observed that DNA damage and repair genes were significantly decreased in the flight samples when compared to the post-flight and ground samples. This is different to the observation from the study by Baio et al. (2018) where both the neonatal and adult CPCs showed increased expression of DNA repair genes, whilst in a study utilizing simulated microgravity on the ground, there is only increased expression of DNA repair genes in neonatal CPCs (Fuentes et al., 2015; Wnorowski et al., 2019). As results from ground simulated microgravity and spaceflight microgravity studies and the effect it has on the cardiovascular system has been conflicting there may be other factors to consider, one of them being the level of radiation that will be considerably higher aboard the ISS than on Earth (Moreno-Villanueva et al., 2017; Wnorowski et al., 2019). The difference in the level of radiation may impact the cell’s response aboard in-flight and on the ground. Molecularly, radiation and microgravity have been shown to have a negative impact on the integrity of DNA. To prevent DNA damage, cells have developed specific mechanisms that allow them to locate and repair DNA lesions. Usually, cells can withstand a moderate level of DNA damage with the help of these repair processes; however, the lack of gravity may unfavorably affect this process that tries and prevent the DNA damage, leading to the accumulation of DNA miss-matched repair (Moreno-Villanueva et al., 2017).

## Cardiac Mechanotransduction

Previously mentioned study by Hughson et al. (2016) suggested that microgravity and radiation cause an increase in stiffness in carotid artery. The carotid artery stiffness was compared between pre- and postflight; it was seen that the stiffness was significantly increased postflight. Studies have shown that under normal gravity condition this increase in stiffness happens with the increase in age. However, this increase in stiffness could already be seen in the participants of the study as a result of the space condition (Hughson et al., 2016). Animal model further confirmed that space environment contributes to higher aortic stiffness, where rats exposed to high energy radiation that simulated the space environment also have significantly higher aortic stiffness over a 6-month period (Soucy et al., 2011). Although the mechanism for the change is different between spaceflight and aging, the magnitude of change is significant. Arterial stiffness has also been believed to be a precursor to atherosclerosis and a marker for increased cardiovascular disease risk such as myocardial infarction (O’Rourke, 1995; Safar et al., 2003; Zieman et al., 2005). The majority of upregulated proteins in the aortic wall are involved in the actin cytoskeleton organization and by identifying the exact molecular mechanism and mechanical regulators associated with arterial stiffness, it can aid in identifying therapeutic intervention (Miotto et al., 2021). Interestingly, the increased stiffness in the carotid artery that is normally seen in the aging cardiovascular system in the population can be simulated by spaceflight (Kawasaki et al., 1987; Lakatta and Levy, 2003; Gepner et al., 2014; Hughson et al., 2016). This process can be utilized in the field of space mechanobiology for more in-depth study of the mechanotransduction responses in the cardiovascular system in relation to cardiovascular diseases.

**FIGURE 1:**
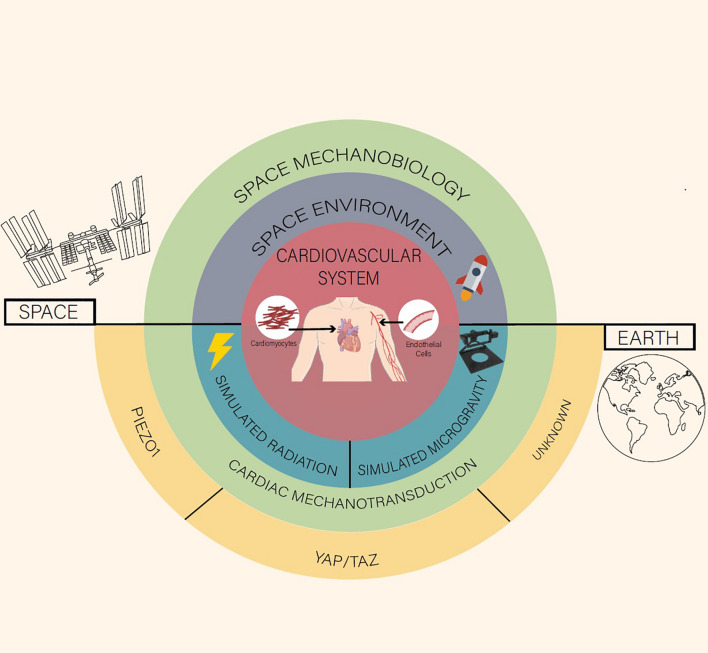
A space mechanobiology approach toward cardiovascular function and disease.

There are a number of proteins that are sensitive and responsive to changes in the mechanical environment and looking at these key proteins could elucidate the relationship between cell function and their responses to these mechanical cues. By looking at the expressions of these proteins, it can then be used as a measure of mechanotransduction (Chin et al., 2019).

The Yes-associated protein (YAP) and its paralog TAZ (transcription co-activator) have been demonstrated as major regulators of cardiac cell survival, proliferation, and differentiation (Xin et al., 2011; von Gise et al., 2012; Lin et al., 2014). It has been found that activation of YAP improves cardiac regeneration (Xin et al., 2013). Furthermore, when cardiomyocyte-specific activated YAP is overexpressed, there is an increase in the proliferation of cardiomyocytes which leads to a cardiac overgrowth in neonatal mice (Xin et al., 2011). In supporting this, it has been found that YAP protein can be detected in neonatal hearts; however, with age, the expression decreases, while the phosphorylation of YAP increases with age (von Gise et al., 2012). However, nuclear YAP expression that is usually missing in adult cardiomyocytes shows in infarcted cardiac tissue at the border of the infarcted region. The reason for this could be a result of the increased stiffness in the ECM that is exhibited in the infarcted area (Mosqueira et al., 2014). Interestingly, both spaceflight and simulated microgravity has been shown to upregulate YAP1 expression in adult CPCs and downregulation in neonatal CPCs (Baio et al., 2018; Camberos et al., 2019). This indicates that microgravity can induce functions and responses to damage on the heart.

Another key cardiac mechanotransducer associated with the mechanical stretching of cardiac tissues is Piezo1, which is a membrane mechanosensor. Activation of Piezo1 occurs due to the vascular shear stress in the human body, which is derived through the effects of heart contraction (Beech and Kalli, 2019). Recently it has been shown that Piezo1 in cardiomyocytes is triggered by cell stretching (Guo et al., 2020), which indicates that Piezo1 plays a vital part as an ion channel contributing to the feedback of the mechano-electric response of the cardiac cells utilizing its Ca^2+^ transient control during cardiac cell stretching. Another study confirmed that the Piezo1 channel directly converts the mechanical stretch of cardiomyocytes into calcium ion (Ca^2+^) and reactive oxygen species (ROS) signaling, and when Piezo1 is overexpressed or deleted, it resulted in heart dysfunction (Jiang et al., 2021).

It is evident that mechanotransduction in the form of mechanical cues, ECM and mechanical load in the form of gravity plays a pivotal role in cardiac homeostasis beyond just YAP and PIEZO. The current gap lies in the fundamental drivers of these functions and mechanisms remains unresolved.

## Discussion

The human heart is a vital organ that pumps blood throughout the body using the circulatory system to supply nutrients and remove waste from the tissues. As it is a vital part of the human body, it is important that the issue of cardiovascular health and diseases are understood and addressed. The complexity of the cellular anatomy of the human heart makes it challenging to develop a clinically relevant model. Therefore, future effort to address this could include the use of novel *in vitro* models, including organs on a chip to mature cells (Chen et al., 2013; Ren et al., 2013; Sharma et al., 2021). Even with an established *in vitro* and/or *in vivo* model, to initiate the onset of CVD conditions remains a challenge. As outlined in this mini review, cardiac mechanotransduction plays an important role in not only understanding the cardiovascular system, but also in the maturation and the functional response of the cardiac tissue. It has been demonstrated in studies how cardiac mechanosensing regulators including YAP and PIEZO1 regulate cardiac tissue function and dysfunction, including cardiac cell survival, proliferation, differentiation and responses to mechanical stimuli (Xin et al., 2011; von Gise et al., 2012; Lin et al., 2014; Beech and Kalli, 2019; Guo et al., 2020; Jiang et al., 2021). The main effects of microgravity and radiation on the cardiovascular system has been demonstrated to show changes can be seen from a physiological to a cellular level, including changes to cardiac and endothelial function, increased stiffness of carotid artery, oxidative stress and inflammation as well as altered fluid distribution and decreased calcium recycling rate. As further highlighted in this mini-review the complexity and cross-talk between different mechanosensors and regulators remain elusive and reinforce the opportunities for cardiac and space biologists in investigating this interface of cell biology and biomedical engineering.

A key element that was not discussed in this mini review is the technologies to conduct microgravity and/or simulated microgravity research but there are clear advancements in this area made available for researchers. Whilst there have been several studies in space on the cardiac responses and genetic and epigenetic profiling on the effects of microgravity, this sets the foundation and the stage to examine the underlying cellular mechanisms of the cardiovascular system. More comparison between experiments on Earth and ISS needs to be done to validate the simulations done on the ground and open more doors to research that simulate the space conditions which will allow us to look into space mechanobiology with greater control. Furthermore, as demonstrated in this mini review, the majority of research shown on cardiac cells have been done on microgravity and radiation independently; however, there are limited studies that combine these two external stressors on cells of cardiovascular origin. It is also important to note that the effects of microgravity and radiation has been varied depending on factors such as cell type, specific bioreactor/cultured environment used and the type of exposure.

The unwavering question of whether microgravity or radiation or the dual effects have on cardiac function remains open for discovery and ultimately both of these conditions are a new type of extreme environment in which researchers can uncover knowledge from cellular to organ level. As shown in this mini review, there is still a great amount of unknown to the underlying mechanisms of cardiac function and CVD onsets, and whilst there exist several novel and innovative approaches toward advancing this understanding, gap in knowledge remains. The research outlined in this mini review provides a glimpse into what can be achieved through microgravity research in advancing cardiac knowledge and with current CVD death toll still remaining high, it may be time to take an out of this world approach toward CVD.

## Author Contributions

CB wrote the manuscript. SN contributed to the section “The Effect of Microgravity on the Cardiovascular System.” MF, G-JL, RM, and NH contributed to the section “The Effect of Radiation on the Cardiovascular System.” CG, BM, KP, and JC initiated, conceptualized the review, and edited the manuscript. All authors have read and agreed to the published version of the manuscript.

## Conflict of Interest

The authors declare that the research was conducted in the absence of any commercial or financial relationships that could be construed as a potential conflict of interest.

## Publisher’s Note

All claims expressed in this article are solely those of the authors and do not necessarily represent those of their affiliated organizations, or those of the publisher, the editors and the reviewers. Any product that may be evaluated in this article, or claim that may be made by its manufacturer, is not guaranteed or endorsed by the publisher.

## Abbreviations

CVD: cardiovascular disease
ECM: extracellular matrix
ISS: International Space Station
CPCs: cardiac progenitor cells
hiPSC-CMs: human induced pluripotent stem cells
CGR: cosmic galactic rays
SPE: Solar Particle Events
EVA: Extra Vehicular Activities
LET: Linear Energy Transfer
ECs: endothelial cells
DSBs: DNA double strand breaks
YAP: yes-associated protein
TAZ: transcription co-activator
Ca^2+^: calcium ion
ROS: reactive oxygen species
RPM: random positioning machine
RWV: rotating wall vessel.
